# Taxonomy and Phylogeny of Four New Species in *Absidia* (Cunninghamellaceae, Mucorales) From China

**DOI:** 10.3389/fmicb.2021.677836

**Published:** 2021-08-04

**Authors:** Tong-Kai Zong, Heng Zhao, Xiao-Ling Liu, Li-Ying Ren, Chang-Lin Zhao, Xiao-Yong Liu

**Affiliations:** ^1^Key Laboratory for Forest Resources Conservation and Utilization in the Southwest Mountains of China, Ministry of Education, Southwest Forestry University, Kunming, China; ^2^State Key Laboratory of Mycology, Institute of Microbiology, Chinese Academy of Sciences, Beijing, China; ^3^College of Life Science, University of Chinese Academy of Sciences, Beijing, China; ^4^College of Plant Protection, Jilin Agricultural University, Changchun, China; ^5^College of Biodiversity Conservation, Southwest Forestry University, Kunming, China

**Keywords:** morphology, molecular phylogeny, taxonomy, Mucoromycetes, Mucoromycota

## Abstract

Four new species within the genus *Absidia*, *A. globospora*, *A. medulla*, *A. turgida*, and *A. zonata*, are proposed based on a combination of morphological traits, physiological features, and molecular evidences. *A. globospora* is characterized by globose sporangiospores, a 1.0- to 3.5-μm-long papillary projection on columellae, and sympodial sporangiophores. *A. medulla* is characterized by cylindrical to oval sporangiospores, a 1.0- to 4.5-μm-long bacilliform projection on columellae, and spine-like rhizoids. *A. turgida* is characterized by variable sporangiospores, up to 9.5-μm-long clavate projections on columellae, and swollen top of the projection and inflated hyphae. *A. zonata* is characterized by cylindrical to oval sporangiospores, a 2.0- to 3.5-μm-long spinous projection on columellae, and as many as eight whorled sporangiophores. Phylogenetic analyses based on sequences of internal transcribed spacer rDNA and D1–D2 domains of LSU rDNA support the novelty of these four species within the *Absidia*. All new species are illustrated, and an identification key to all the known species of *Absidia* in China is included.

## Introduction

The genus *Absidia* Tiegh. (Cunnighamellaceae, Mucorales, Mucoromycetes, Mucoromycota) was proposed by van Tieghem (1876). *Absidia* members are ubiquitous in soil and also often associated with warm decaying plant matter, such as compost heaps. Some *Absidia* can be used to produce chitin, chitosan, and chitooligosaccharides (Kaczmarek et al., 2019) and hydrocortisone (Chen et al., 2020). *Absidia* species typically have sporangiophores arising from stolons, rhizoids never opposite the sporangiophores, pyriform sporangia and their deliquescent wall, obvious apophyses, a septum beneath the sporangium, and zygospores surrounded by appendages from the suspensors (Hoffmann et al., 2007; Hoffmann, 2010).

The classification and circumscription of the *Absidia* have been debated since it was described. According to zygospore morphology, Hesseltine and Ellis (1964) divided *Absidia* into two subgenera, the subgenus *Absidia* and the subgenus *Mycocladus* (Beauverie) Hesselt. & J.J. Ellis. Different from the former, the subgenus *Mycocladus* does not form any appendages from the suspensors of zygospores. This classification framework was followed by Schipper (1990), who further divided the subgenus *Absidia* into six groups. However, this kind of delimitation are not accepted nowadays, and six genera are synonymized with the genus *Absidia* instead (Hawksworth et al., 1995). They are *Tieghemella* Berl. & De Toni 1888, *Mycocladus* Beauverie 1900, *Lichtheimia* Vuill 1903, *Proabsidia* Vuill 1903, *Pseudoabsidia* Bainier 1903, and *Protoabsidia* Naumov 1935. Among these synonyms, *Lichtheimia*, *Mycocladus*, *Pseudoabsidia*, and *Protoabsidia* lack appendages.

Recently, a combined study of molecular phylogenetics, morphology, and physiology has provided a more reliable delimitation among *Absidia* species (Hoffmann et al., 2007), where *Absidia* was classified into three groups: (1) the thermotolerant species with an optimal growth temperature of 37–45°C, which were then transferred into the genus *Lichtheimia* (Hoffmann et al., 2009a); (2) the mesophilic species with an optimal growth temperature of 25–34°C, which have been accepted up to now as *Absidia sensu stricto*; and (3) the mycoparasitic species, potential to parasitize other mucoralean hosts with optimal growth temperatures below 30°C, which were then transferred into the genus *Lentamyces* Kerst. Hoffm. & K. Voigt (Hoffmann and Voigt, 2008). Currently, 37 species have been reported worldwide in *Absidia* (Hesseltine and Ellis, 1961, 1964, 1966; Ellis and Hesseltine, 1965, 1966; Index Fungorum^1^). Among these species, 13 were reported in the last decade using the strategy of combing morphology, physiology, and phylogeny: *Absidia caatinguensis* D.X. Lima and A.L. Santiago, *Absidia cornuta* D.X. Lima, C.A. de Souza, H.B. Lee, and A.L. Santiago; *Absidia jindoensis* Hyang B. Lee, and T.T.T. Nguyen; *Absidia koreana* Hyang B. Lee, Hye W. Lee, and T.T. Nguyen; *Absidia multispora* T.R.L. Cordeiro, D.X. Lima, Hyang B. Lee, and A.L. Santiago; *Absidia panacisoli* T. Yuan Zhang, Ying Yu, He Zhu, S.Z. Yang, T.M. Yang, Meng Y. Zhang, and Yi X. Zhang; *Absidia pararepens* Jurjeviæ, M. Kolaøík, and Hubka; *Absidia pernambucoensis* D.X. Lima, C.M. Souza-Motta, and A.L. Santiago; *Absidia saloaensis* T.R.L. Cordeiro, D.X. Lima, Hyang B. Lee, and A.L. Santiago; *Absidia stercoraria* Hyang B. Lee, H.S. Lee, and T.T.T. Nguyen; *Absidia terrestris* Rosas de Paz, Dania García, Guarro, Cano, and Stchigel; *Absidia bonitoensis* C.L. Lima, D.X. Lima, Hyang B. Lee, and A.L. Santiago; and *Absidia ovalispora* H. Zhao and X.Y. Liu (Ariyawansa et al., 2015; Li et al., 2016; Crous et al., 2018, 2020; Wanasinghe et al., 2018; Zhang et al., 2018; Cordeiro et al., 2020; Lima et al., 2020; de Lima et al., 2021; Zhao et al., 2021), and nine species have been recorded in China (Zhang et al., 2018; Zheng and Liu, 2018; Zhao et al., 2021).

Recently, seven strains of *Absidia* were collected from China but could not be assigned to any described species. Herein, morphological, physiological, and molecular phylogenetics [internal transcribed spacer (ITS) and D1–D2 domains of LSU rDNA] are presented to support them to four new species in *Absidia sensu stricto*, and consequently, a revised synoptic key to all the 13 known species of *Absidia* in China is provided.

## Materials and Methods

### Isolation and Strains

Strains were isolated from the soil collected in Hubei province, Shanxi province, Xinjiang province, and Yunnan province, China. Soil samples (1 g) were suspended in 100 mL sterilized water and shaken vigorously. Then, a 100 μL of the suspension was added onto a potato dextrose agar (PDA; Benny, 2008) plate with antibiotics streptomycin sulfate (100 mg/mL) and ampicillin (100 mg/mL). The plate was incubated at 27°C and examined daily with a stereo microscope (SMZ1500, Nikon Corporation, Japan). Upon the presence of colonies, a single colony was picked and transferred to new PDA plates. Living cultures were deposited in the China General Microbiological Culture Collection Center, Beijing, China (CGMCC). Dried cultures were deposited in the Herbarium Mycologicum Academiae Sinicae, Beijing, China (HMAS).

### Morphology and Growth Experiments

Pure cultures were established in triplicate, respectively, with malt extract agar (MEA; Benny, 2008), modified synthetic mucor agar (SMA; Zheng and Chen, 2001), and PDA plates. For morphological observation, they were incubated at 27°C for 4–7 days and examined daily under a microscope (Axio Imager A2, Carl Zeiss Microscopy, Germany). For determining maximum growth temperatures, pure cultures were initially incubated at 32°C for 4 days, and then the incubation temperature was adjusted until the colonies stopped growing. The color of colonies was designated according to Ridgway (1912).

### DNA Extraction, Polymerase Chain Reaction Amplification, and Sequencing

Mycelia were grown at 27°C for 5 days on PDA plates, and then cell DNAs were extracted using a kit (GO-GPLF-400, GeneOnBio Corporation, Changchun, China). The ITS and D1–D2 domain of LSU rDNA were amplified with primer pairs NS5M and LR5M (Wang et al., 2014). The polymerase chain reaction (PCR) procedure was as follows: an initial temperature at 95°C for 5 min; then 30 cycles of denaturation at 95°C for 20 s, annealing at 55°C for 60 s, and extension at 72°C for 60 s; and finally an extra extension at 72°C for 10 min. PCR products were purified and then sequenced with primers ITS5 (White et al., 1990) and LR5M at BGI Tech Solutions Beijing Liuhe Co., Limited, Beijing, China. All newly generated sequences were deposited in GenBank and National Microbiology Data Center (NMDC,^2^ Table 1).

**TABLE 1:** Species, strains, and GenBank/NMDC accession numbers used in this study.

**Species**	**Strains**	GenBank/NMDC* accession no.	
		**ITS**	**LSU**
*Absidia anomala*	CBS 125.68	MH859085	NG058562
*Absidia bonitoensis*	URM 7889	MN977786	MN977805
*Absidia caatinguensis*	URM 7156	KT308169	KT308171
*Absidia coerulea*	CBS 101.36	MH855718	MH867230
*Absidia californica*	CBS 314.78	MH861141	MH872902
*Absidia cornuta*	URM 6100	MN625256	MN625255
*Absidia cuneospora*	CBS 101.59		NG058559
*A. cuneospora*	FSU 5890	EF030524	
*Absidia cylindrospora*	FSU 906	AY944889	
*A. cylindrospora*	CBS 100.08		JN206588
*Absidia fusca*	CBS 102.35	NR103625	NG058552
*Absidia glauca*	CBS 129233	MH865253	MH876693
*A. glauca*	CBS 127122	MH864429	MH875867
Absidia globospora*	**CGMCC 3.16031**	MW671537/NMDCN0000JB7*	MW671544/NMDCN0000JB0*
A. globospora*	**CGMCC 3.16035**	MW671538/NMDCN0000JB8*	MW671545/NMDCN0000JB1*
A. globospora*	**CGMCC 3.16036**	MW671539/NMDCN0000JB9*	MW671546/NMDCN0000JB2*
*Absidia heterospora*	SHTH021	JN942683	JN982936
*Absidia jindoensis*	CNUFC-PTI1-2	MF926623	MF926617
*Absidia koreana*	EML-IFS45-1	KR030062	KR030056
*A. koreana*	EML-IFS45-2	KR030063	KR030057
*Absidia macrospora*	FSU 4746	AY944882	EU736303
Absidia medulla*	**CGMCC 3.16034**	MW671542/NMDCN0000JBC*	MW671549/NMDCN0000JB5*
A. medulla*	**CGMCC 3.16037**	MW671543/NMDCN0000JBD*	MW671550/NMDCN0000JB6*
*Absidia multispora*	URM 8210	MN953780	MN953782
*Absidia ovalispora*	CGMCC 3.16018	MW264071	MW264130
*Absidia panacisoli*	SYPF 7183	MF522181	MF522180
*Absidia pararepens*	CCF 6352	MT193669	MT192308
*Absidia pernambucoensis*	URM 7219	MN635568	MN635569
*Absidia pseudocylindrospora*	CBS 100.62		MH869688
*A. pseudocylindrospora*	FSU5894	EF030526	
*Absidia psychrophilia*	FSU 4745	AY944874	EU736306
*Absidia repens*	CBS 115583	NR103624	HM849706
*Absidia saloaensis*	URM 8209	MN953781	MN953783
*Absidia spinosa*	FSU 551		EU736307
*Absidia stercoraria*	EML-DG8-2	KU168829	KT921999
*Absidia terrestris*	FMR 14989		LT795005
*A. terrestris*	FMR 15024	LT795004	
Absidia turgida*	**CGMCC 3.16032**	MW671540/NMDCN0000JBA*	MW671547/NMDCN0000JB3*
Absidia zonata*	**CGMCC 3.16033**	MW671541/NMDCN0000JBB*	MW671548/NMDCN0000JB4*
*Chlamydoabsidia padenii*	CBS 172.67	JN206294	NG070364
*Halteromyces radiatus*	CBS 162.75	JN206290	NG057938
*Cunninghamella elegans*	CBS 167.53	JN205882	HM849700
*Cunninghamella blakesleeana*	CBS 782.68	JN205869	MH870950

*All strains of the four new species are in bold font, and their sequences are deposited at National Microbiology Data Center (NMDC).

### Phylogenetic Analyses

The software platform Geneious 8.1^3^ was used to assemble and proofread DNA sequences. All the sequences were realigned using AliView version 3.0 (Larsson, 2014). The sequence alignments and phylogenetic trees were deposited at TreeBase (submission ID 27734). Sequences of *Cunninghamella elegans* and *Cunninghamella blakesleeana* retrieved from GenBank were used as outgroups in the ITS and LSU analyses following Hoffmann et al. (2007).

Phylogenetic analyses were carried out using maximum parsimony (MP), maximum likelihood (ML), and Bayesian inference (BI). MP phylogenetic analyses followed Zhao and Wu (2017), and the tree construction was performed in PAUP^∗^ version 4.0b10 (Swofford, 2002). All characters were equally weighted, and gaps were treated as missing data. Trees were inferred using the heuristic search option with TBR branch swapping and 1,000 random sequence additions. Max-trees were set to 5,000; branches of zero length were collapsed, and all parsimonious trees were saved. Clade robustness was assessed using a bootstrap analysis with 1,000 replicates (Felsenstein, 1985). Descriptive tree statistics tree length (TL), consistency index (CI), retention index (RI), rescaled CI (RC), and homoplasy index (HI) were calculated for each maximum parsimonious tree generated.

Maximum likelihood phylogenetic analyses were conducted with raxmlGUI 2.0 beta (Edler et al., 2020). A general time reversible model was used with a gamma-distributed rate variation (GTR + G) and 1,000 bootstrap replicates.

Bayesian inference phylogenetic analyses was calculated with MrBayes 3.2.7a by a general time-reversible model with an estimate of the proportion of invariant sites and a gamma distribution for variable rates across sites (GTR + I + G; Ronquist et al., 2012). Four Markov chains were run simultaneously from random starting trees, for 2,400,000 generations (ITS) or 300,000 generations (LSU). Trees were sampled every 100 generations. The chains stopped once the average standard deviation of split frequencies decreased lower than 0.01. The first one-fourth generations were discarded as burn-in. A majority rule consensus tree of all remaining trees was calculated. Branches were considered as significantly supported if they received ML bootstrap > 75%, MP bootstrap > 75%, or Bayesian posterior probabilities > 0.95.

## Results

### Phylogenetic Analyses

**FIGURE 1:**
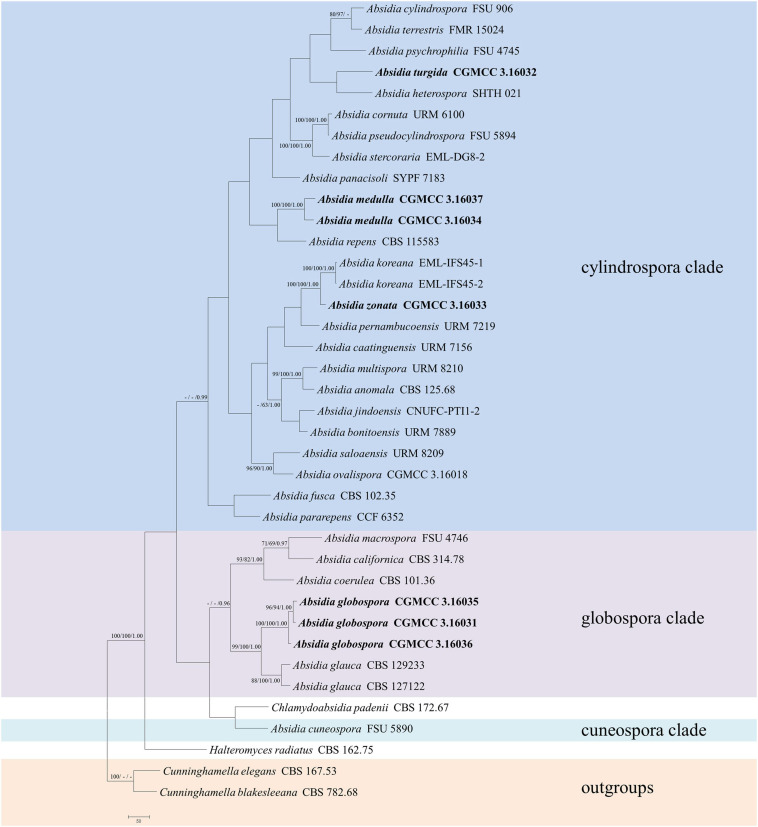
The maximum parsimony strict consensus tree illustrating the phylogeny of four new species of *Absidia* and related species in Cunninghamellaceae based on ITS sequences. *Cunninghamella elegans* and *Cunninghamella blakesleeana* serve as outgroups. Branches are labeled with maximum likelihood bootstrap values higher than 70%, maximum parsimony bootstrap values higher than 50%, and Bayesian posterior probabilities more than 0.95. The lower left scale represents steps.

The ITS dataset included sequences from 38 strains representing 33 species of *Absidia* and related genera. The dataset had an aligned length of 903 characters, of which 248 characters were constant, 136 were variable and parsimony-uninformative, and 519 were parsimony-informative. MP analyses yielded two equally parsimonious trees (TL = 4163, CI = 0.3394, HI = 0.6606, RI = 0.3446, RC = 0.1170). At the end of the inference, the average standard deviation of split frequencies was 0.009990. All BI, ML, and MP phylogenetic trees resulted in similar topologies. The phylogram (Figure 1) consists of three clades, although with relatively low support values: (1) except the *A. pararepens* and *A. bonitoensis*, all members in the cylindrospora clade produce cylindric sporangiospores; (2) all members in the globospora clade produce globose sporangiospores; and (3) the *Absidia cuneospora* G.F. Orr & Plunkett separately groups as a cuneospora clade, forming conical sporangiospores (Orr and Plunkett, 1959).

**FIGURE 2:**
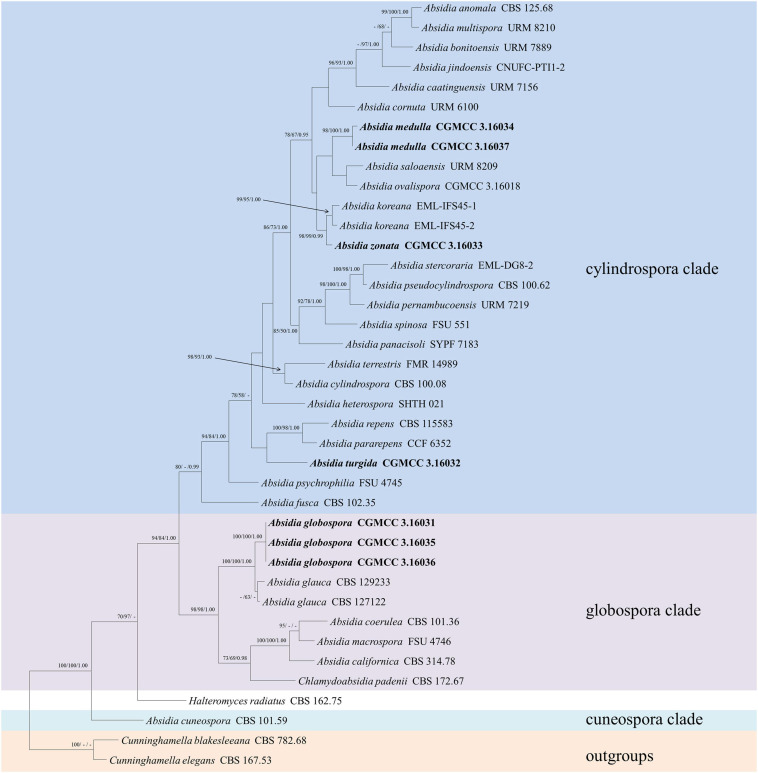
The maximum parsimony strict consensus tree illustrating the phylogeny of four new species of *Absidia* and related species in *Absidia* based on LSU sequences. *Cunninghamella elegans* and *Cunninghamella blakesleeana* serve as outgroups. Branches are labeled with maximum likelihood bootstrap values higher than 70%, maximum parsimony bootstrap values higher than 50%, and Bayesian posterior probabilities more than 0.95. The lower left scale represents steps.

The LSU dataset included sequences from 39 strains representing 34 species within *Absidia*. The dataset had an aligned length of 967 characters, of which 562 characters were constant, 117 were variable and parsimony-uninformative, and 288 were parsimony-informative. MP analyses yielded 20 equally parsimonious trees (TL = 1422, CI = 0.4339, HI = 0.5661, RI = 0.6443, RC = 0.2795). At the end of the inference, the average standard deviation of split frequencies was 0.009767. All BI, ML, and MP phylogenetic trees resulted in similar topologies. The phylogram (Figure 2) consists of three clades, similar to the ITS phylogram (Figure 1) but with relatively high support values, in detail, clade cylindrospora, globospora, and cuneospora with a support of 80/-/0.99, 98/98/1.00, and 100/100/1.00, respectively.

### Taxonomic Treatments

#### *Absidia globospora* T.K. Zong & X.Y. Liu, sp. nov.

**FIGURE 3:**
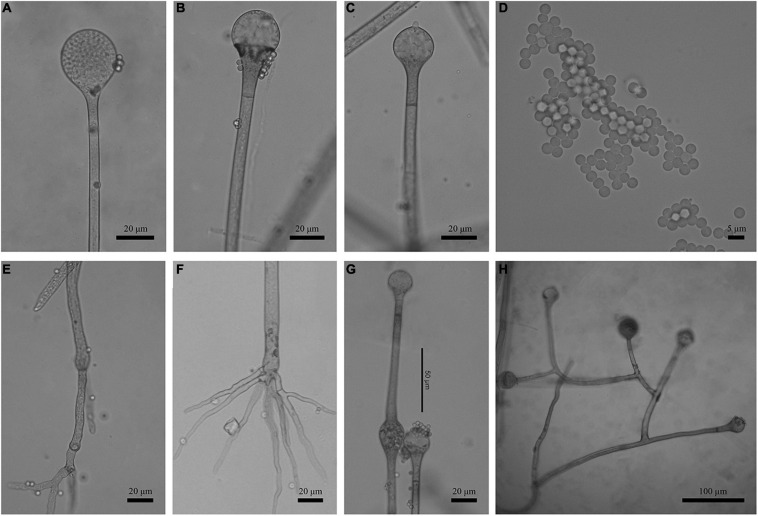
Morphologies of *Absidia globospora* CGMCC 3.16031. **(A)** Sporangium; **(B,C)** columellae; **(D)** sporangiospores; **(E,F)** rhizoids; **(G)** swelling on sporangiospores; **(H)** sympodial sporangiophores. Scale bars: **(A–C,E–G)** 20 μm; **(D)** 5 μm; **(H)** 100 μm.

**FIGURE 4:**
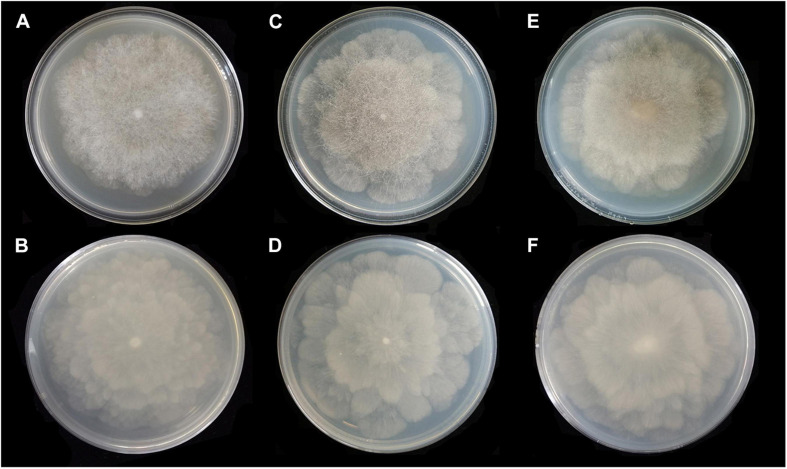
Colonies of *Absidia globospora* CGMCC 3.16031 at 27°C after 6 days on MEA **(A)** obverse, **(B)** reverse; on SMA **(C)** obverse, **(D)** reverse; on PDA **(E)** obverse, and **(F)** reverse.

Fungal names: FN570833 (Figures 3, 4).

Holotype: China. Hubei Province, Shennongjia Forestry District, from soil sample, 20 August 1984, Chen Guiqing (HMAS 249881, living culture CGMCC 3.16031. GenBank: ITS = MW671537, LSU = MW671544. NMDC: ITS = NMDCN0000JB7, LSU = NMDCN0000JB0).

Paratype: China. Shanxi Province, Baoji, Tangyu County, Taibaishan National Forest Park, from soil sample, 11 October 2002, Wang Xuewei (CGMCC 3.16035. GenBank: ITS = MW671538, LSU = MW671545. NMDC: ITS = NMDCN0000JB8, LSU = NMDCN0000JB1); Hubei Province, Shennongjia District, Hubei Shennongjia Forest Ecosystem National Field Scientific Observation and Research Station, from an unknown substrate, 16 October 2002, Wang Xuewei (CGMCC 3.16036. GenBank: ITS = MW671539, LSU = MW671546. NMDC: ITS = NMDCN0000JB9, LSU = NMDCN0000JB2).

Etymology: *globospora* (Lat.) referring to the shape of sporangiospores.

Description: Colonies on MEA, irregularly zonate, attaining 73-mm diameter after 6 days at 27°C, white at first and then elm green (R17) to dark cress green (R31). Hyphae hyaline at first, becoming brown when mature (6.0–)8.0–13.5(–14.5)-μm diameter. Stolons branched, hyaline to brown, smooth, with few septa near the base of sporangiophores (6.0–)7.0–10.5-μm diameter. Rhizoids root-like, branched mostly twice and rarely repeatedly, with a septum at the base. Sporangiophores erect or slightly bent, 1–5 in whorls, unbranched, simple, monopodial or sympodial, hyaline, or brown, with a septum (9.5–)11.0–21.5 μm below apophyses, sometimes a swelling beneath sporangia (45.0–)65.0–350.0(–440.0) × 5.0–8.5(–9.5) μm. Apophyses distinct, slightly pigmented (3.0–)4.0–14.0(–20.0) μm high, 4.0–11.0(–13.5) μm wide at the base, and 11.0–24.0(–26.5) μm wide at the top. Sporangia globose, multispored, deliquescent-walled (20.5–)23.0–50.0(–55.5) × (17.0–)23.0–40.0(–57.0) μm. Columellae hemispherical, hyaline, smooth, sometimes with a 1–3.5 μm papillary projection at the apex, 12.5–33.5(–48.5) × (8.5–)10.0–31.5(–46.5) μm. Collars present or absent, but indistinct if present. Sporangiospores globose, hyaline, smooth, 3.0–4.0(–4.5) × 2.5–3.5(–4.0) μm. Chlamydospores absent. Zygospores not observed.

Media and temperatures: Colonies on SMA, flower-shaped, attaining 74-mm diameter after 6 days at 27°C, white at first and then olive-citrine (R16) to Kronberg’s green (R31). Colonies on PDA, flower-shaped, attaining 73-mm diameter after 6 days at 27°C, white at first and then cossack green (R6) to cerro green (R5). No growth at 29°C.

#### *Absidia medulla* T.K. Zong & X.Y. Liu, sp. nov.

**FIGURE 5:**
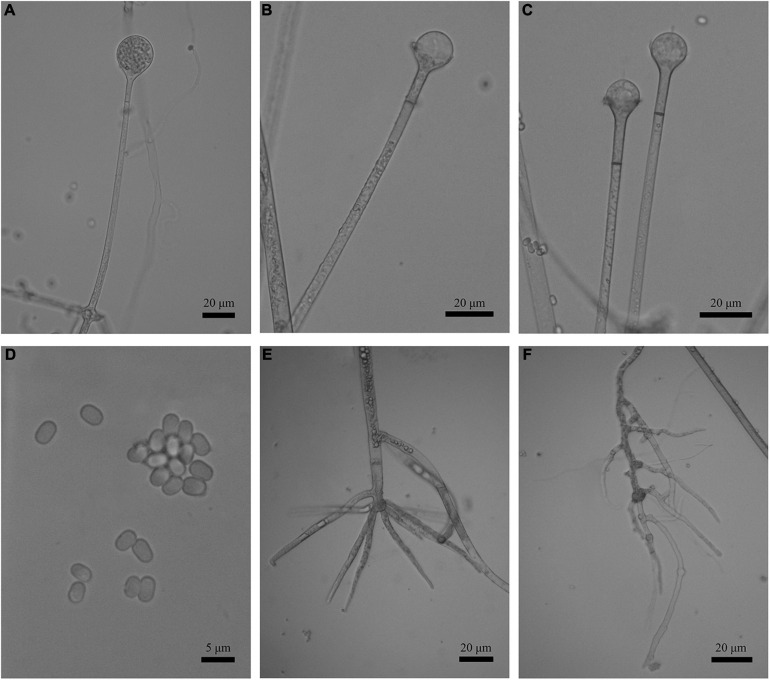
Morphologies of *Absidia medulla* CGMCC 3.16034. **(A)** Sporangium; **(B,C)** columellae; **(D)** sporangiospores; **(E,F)** rhizoids. Scale bars: **(A–C,E,F)** 20 μm; **(D)** 5 μm.

**FIGURE 6:**
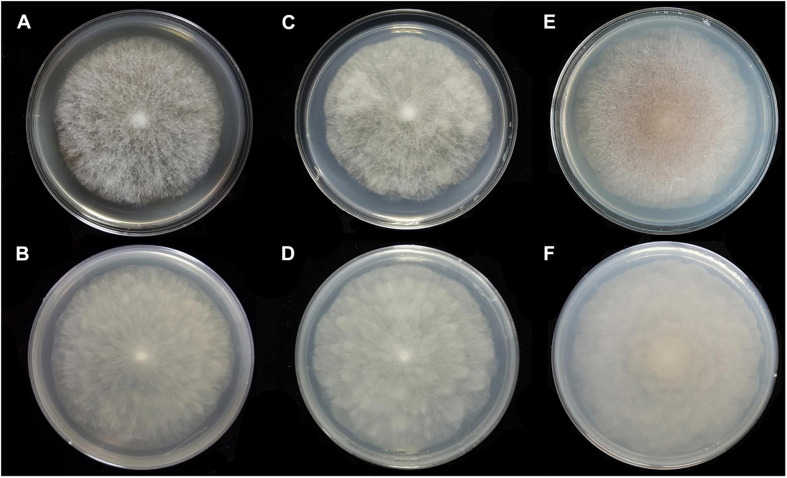
Colonies of *Absidia medulla* CGMCC 3.16034 at 27°C after 5 days on MEA **(A)** obverse, **(B)** reverse; on SMA **(C)** obverse, **(D)** reverse; after 7 days on PDA **(E)** obverse, and **(F)** reverse.

**FIGURE 7:**
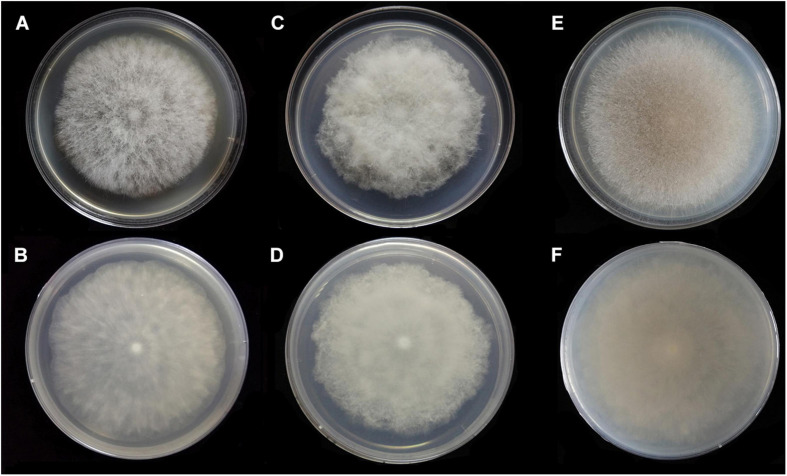
Colonies of *Absidia medulla* CGMCC 3.16037 at 27°C after 5 days on MEA **(A)** obverse, **(B)** reverse; after 6 days on SMA **(C)** obverse, **(D)** reverse; after 5 days on PDA **(E)** obverse, and **(F)** reverse.

Fungal names: FN570836 (Figures 5–7).

Holotype: China. Yunnan Province, Xishuangbanna Dai Autonomous Prefecture, Xishuangbanna, from soil sample, 16 June 1992, Hu Fumei (HMAS 249884, living culture CGMCC 3.16034. GenBank: ITS = MW671542, LSU = MW671549. NMDC: ITS = NMDCN0000JBC, LSU = NMDCN0000JB5).

Paratype: China. Yunnan Province, Kunming, Yunnan Nationalities Village, from soil sample, 25 August 1995, Guo Yinglan (CGMCC 3.16037. GenBank: ITS = MW671543, LSU = MW671550. NMDC: ITS = NMDCN0000JBD, LSU = NMDCN0000JB6).

Etymology: *medulla* (Lat.) referring to the spine-like shape of rhizoids.

Description: Colonies on MEA, regularly zonate, attaining 74-mm diameter after 5 days at 27°C, white at first and then smoke gray (R46), sparse, but abundantly sporulated. Hyphae hyaline at first, becoming brown when mature, septate in age (5.0–)7.0–15.5-μm diameter. Stolons branched, smooth, with few septa near the base of sporangiophores, 3.5- to 6.5-μm diameter. Rhizoids root-like or spine-like, singly to multiply branched, with a septum at the base. Sporangiophores erect or slightly bent, 1–6 in whorls, unbranched, simple or monopodial, rarely sympodial, hyaline, with a septum 12.5–20.5(–27.5) μm below apophyses (50.0–)75.0–200.0(–220.0) × (2.5–)3.0–6.0(–7.5) μm. Apophyses slightly pigmented, 3.0–8.0(–8.5) μm high, 3.0–5.5(–6.5) μm wide at the base, and 7.5–16.5(–17.5) μm wide at the top. Sporangia globose to pyriform, multispored, deliquescent-walled (12.0–)16.0–30.5(–41.0) × (11.5–)15.0–30.0(–32.5) μm. Columellae hemispherical, hyaline, smooth, generally with a single 1.0- to 4.5-μm-long projection, 8.5–20.5 × 7.0–17.5 μm. Collars present or absent, distinct if present. Sporangiospores cylindrical to oval, hyaline, smooth, 3.0–4.5 × 2.0–3.0(–3.5) μm. Chlamydospores absent. Zygospores not observed.

Media and temperatures: Colonies on SMA, cottony, regularly zonate, attaining 70-mm diameter after 5 days at 27°C, white at first and then pale olive-gray (R51). Colonies on PDA, regularly zonate, attaining 74-mm diameter after 5 days at 27°C, white at first and then snuff brown (R29) to deep olive (R40) in center. No growth at 33°C.

#### *Absidia turgida* T.K. Zong & X.Y. Liu, sp. nov.

**FIGURE 8:**
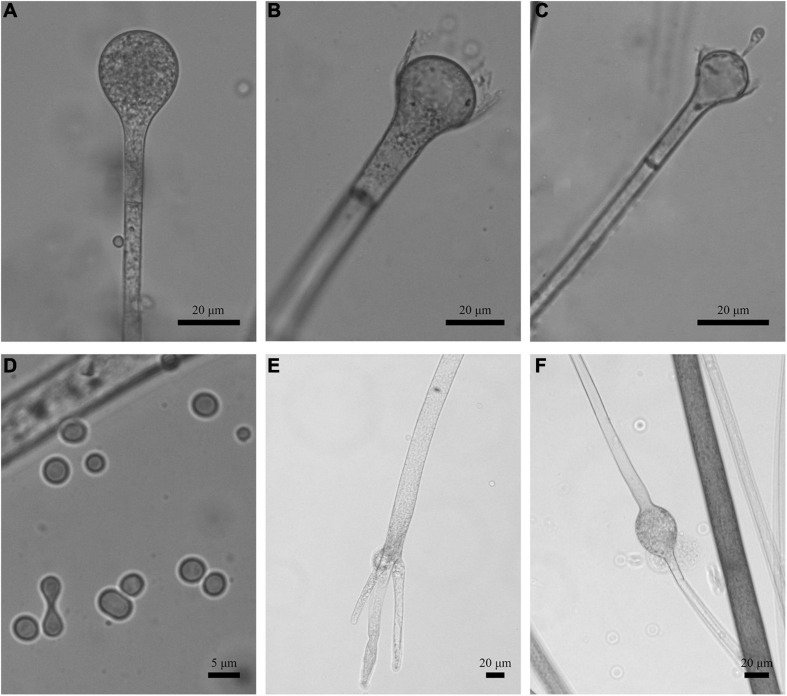
Morphologies of *Absidia turgida* CGMCC 3.16032. **(A)** Sporangium; **(B,C)** columellae; **(D)** sporangiospores; **(E)** rhizoids; **(F)** swollen on hyphae. Scale bars: **(A–C,E,F)** 20 μm; **(D)** 5 μm.

**FIGURE 9:**
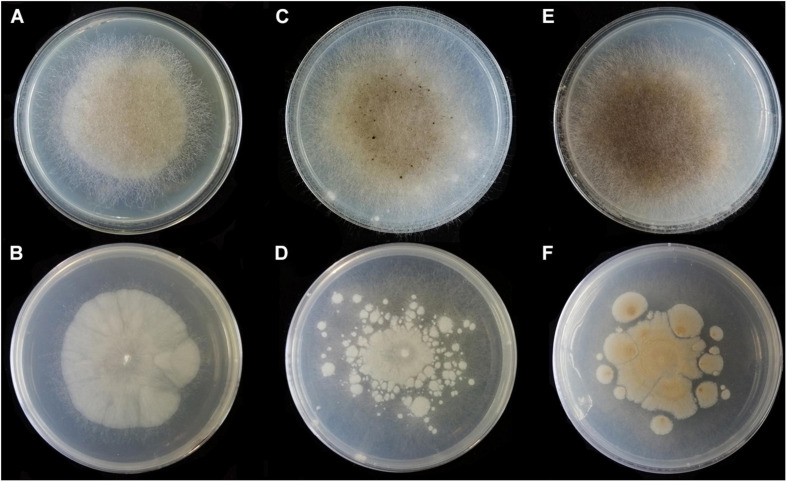
Colonies of *Absidia turgida* CGMCC 3.16032 at 27°C after 12 days on MEA **(A)** obverse, **(B)** reverse; on SMA **(C)** obverse, **(D)** reverse; on PDA **(E)** obverse, and **(F)** reverse.

Fungal names: FN570834 (Figures 8, 9).

Holotype: China. Xinjiang Uygur Autonomous Region, Urumqi, Urumqi County, Xiejiagou Natural Scenic Resort, from soil sample, 7 June 2002, Wang Xuewei (HMAS 249882, living culture CGMCC 3.16032. GenBank: ITS = MW671540, LSU = MW671547. NMDC: ITS = NMDCN0000JBA, LSU = NMDCN0000JB3).

Etymology: *turgida* (Lat.) referring to the swollen hyphae and the inflate projection on columellae.

Description: Colonies on MEA, irregularly radially gaped, attaining 23-mm diameter after 3 days, 35-mm diameter after 7 days, 50-mm diameter after 12 days at 27°C, white at first and then drab gray to drab (R45), sparse, but abundantly sporulated. Hyphae hyaline at first, becoming brown when mature, occasionally swollen, 9.0- to 23.0-μm diameter. Stolons branched, smooth, with few septa near the base of sporangiophores, 8.5- to 16.0-μm diameter. Rhizoids root-like, thick, short or comparatively long, simple or 2–3 branched, with a septum at the base. Sporangiophores erect or slight bent, 1–4 in whorls, unbranched or sometimes simple, hyaline, with a septum (17.0–)21.0–39.5(–43.5) μm below apophyses, 125.0–350.0(–370.0) × (3.5–)4.5–10.0(–11.0) μm. Apophyses distinct, unpigmented (4.5–)5.0–13.5(–16.5) μm high, 3.5–10.0 μm wide at the base, and (10.0–)11.0–22.0(–23.5) μm wide at the top. Sporangia globose to pyriform, multispored, deliquescent-walled, 20.5–42.5 × 20.0–41.5(–46.0) μm. Columellae mostly hemispherical, sometimes conical, hyaline, smooth, with a single clavate projection, up to 9.5 μm in length, with a bulbous swelling at top (13.0–)14.5–25.0(–26.5) × (10.0–)11.5–21.5 μm. Collars present or absent, distinct if present. Sporangiospores variable, globose, cylindrical or irregular, hyaline, smooth, 4.0–5.0(–6.5) × 3.0–4.0 μm when cylindrical, 3.5–4.5 × 3.0–4.0 μm or 2.0- to 2.5-μm diameter when globose. Chlamydospores absent. Zygospores not observed.

Media and temperatures: Colonies on SMA, sporadic, nebula-shaped, attaining 22-mm diameter after 3 days, 32-mm diameter after 7 days, 47-mm diameter after 12 days at 27°C, white at first and then pale drab-gray to light cinnamon-drab (R45). Colonies on PDA, irregularly tree ring-shaped, attaining 21-mm diameter after 3 days, 32-mm diameter after 7 days, 44-mm diameter after 12 days at 27°C, growing slowly when aerial hyphae reaching the lid of the petri dish, white at first and then drab to hair brown (R45). No growth at 33°C.

#### *Absidia zonata* T.K. Zong & X.Y. Liu, sp. nov.

**FIGURE 10:**
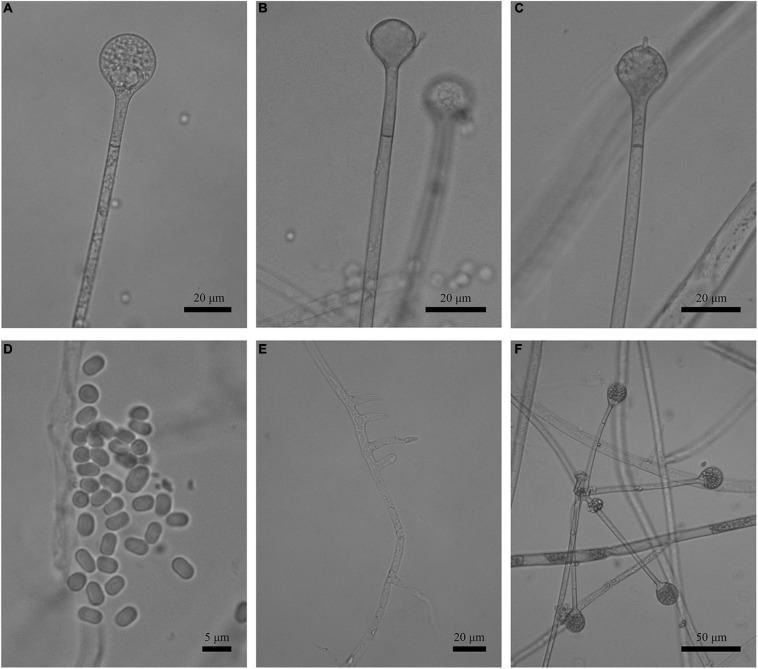
Morphologies of *Absidia zonata* CGMCC 3.16033. **(A)** Sporangium; **(B,C)** columellae; **(D)** sporangiospores; **(E)** rhizoids; **(F)** verticillately branched sporangiophores. Scale bars: **(A–C,E)** 20 μm; **(D)** 5 μm; **(F)** 50 μm.

**FIGURE 11:**
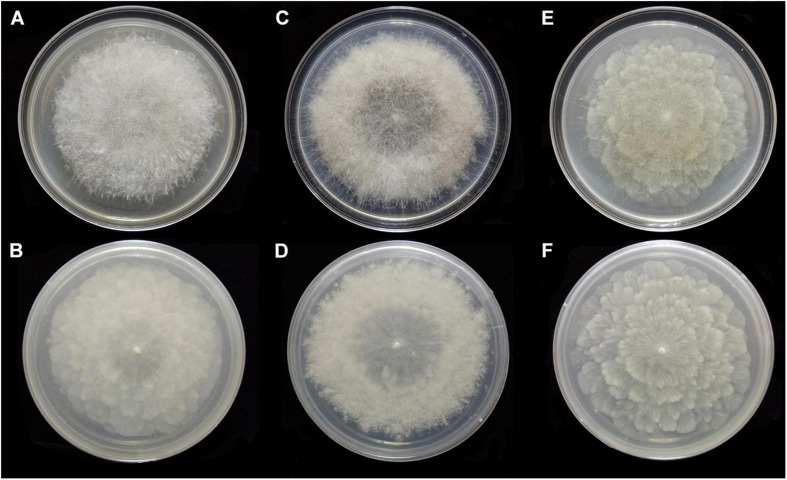
Colonies of *Absidia zonata* CGMCC 3.16033 at 27°C after 8 days on MEA **(A)** obverse, **(B)** reverse; on SMA **(C)** obverse, **(D)** reverse; after 7 days on PDA **(E)** obverse, and **(F)** reverse.

Fungal names: FN570835 (Figures 10, 11).

Holotype: China. Beijing (39°57′58′′N, 116°11′43′′E), from soil sample, 31 December 2019, Liu Xiaoyong (HMAS 249883, living culture CGMCC 3.16033. GenBank: ITS = MW671541, LSU = MW671548. NMDC: ITS = NMDCN0000JBB, LSU = NMDCN0000JB4).

Etymology: *zonata* (Lat.) referring to the zonate colony.

Description: Colonies on MEA, regularly concentric ring zonate, attaining 69-mm diameter after 8 days at 27°C, white at first and then smoke gray (R46). Hyphae hyaline at first, becoming brown when mature, 5.0- to 10.5-μm diameter. Stolons branched, smooth, with few septa near the base of sporangiophores, 4.0- to 8.0-μm diameter. Rhizoids root-like or tentaculiform, simple or 2–3 branched, with a septum at the base. Sporangiophores erect or slightly bent, 1–5(–8) in whorls, unbranched, sometimes simple, rarely monopodial, hyaline, with a septum 16.0–26.5 μm below apophyses (44.0–)55.0–180.0(–280.0) × 2.5–5.5(–6.0) μm. Apophyses distinct, slightly pigmented, 3.0–8.0(–8.5) μm high, 3.0–5.5(–6.5) μm wide at the base, and 7.5–16.5(–17.5) μm wide at the top. Sporangia globose to pyriform, multispored, deliquescent-walled, 14.0–27.0 × 12.5–26.5 μm. Columellae hemispherical, hyaline, smooth, generally presenting a single spinous projection, up to 2.0–3.5 μm in length, 9.5–19.0 × (6.0–)7.5–14.5(–16.5) μm. Collars present or absent, distinct if present. Sporangiospores mostly cylindrical, sometimes oval, hyaline, smooth, 3.5–4.5(–6.0) × 2.0–3.0(–3.5) μm. Chlamydospores absent. Zygospores not observed.

Media and temperatures: Colonies on SMA, rough around the edges, concentric ring-shaped, attaining 69-mm diameter after 8 days at 27°C, white. Colonies on PDA, regularly wavy zonate, attaining 72-mm diameter after 7 days at 27°C, white at first and then lime green (R31). No growth at 38°C.

Key to the known species of *Absidia* in China

1. Sporangiospores typically globose; Colonies greenish ……………………………2

1. Sporangiospores typically cylindrical, oval, or other shaped; Colonies not greenish………………………3

2. Maximum temperatures below 30°C; Sporangiophores not reaching 10 μm in width; Sporangia rarely reaching 55-μm diameter.………………….………*A. globospora*

2. Maximum temperatures above 30°C; Sporangiophores reaching 12 μm in width; Sporangia mostly 50- to 60-μm diameter.……………………………*A. glauca*

3. Columellae without distinct apical projections ……………………………………*A. heterospora*

3. Columellae with apical projections ……………………………4

4. Sporangiospores variable, sometimes irregular in shape and size…………………5

4. Sporangiospores invariable, always regular …………………………6

5. Hyphae without swelling, <9-μm diameter; sporangiophores sometimes simple, more often monopodial or verticillate; columellae sometimes with a short projection…………………………………*Absidia idahoensis*

5. Hyphae occasionally swelling, >9-μm diameter; Sporangiophores unbranched or sometimes simple; columellae always with a projection up to 9 μm in length ………………………………………*A. turgida*

6. Abundant secondary sporangia in older cultures ………………………*A. repens*

6. No abundant secondary sporangia in older cultures ………………………………7

7. Sporangiophores never in pairs or in whorls ……………………………*A. panacisoli*

7. Sporangiophores in pairs or in whorls ………………………………………………8

8. Sporangiophores no more than 6 in whorls ………………………………………9

8. Sporangiophores as many as 7–11 in whorls ……………………………………11

9. Maximum temperatures below 35°C. ……………………………………*A. medulla*

9. Maximum temperatures above 35°C ……………………………………………10

10. Sporangiophores up to 6 in whorls, occasionally swollen below the sporangia; Collar absent; Sporangiospores ovoid to ellipsoid….…………………*A. ovalispora*

10. Sporangiophores up to 4 in whorls, no swollen; Collar always present; Sporangiospores cylindrical………………………………………*A. cylindrospora*

11. Rhizoids typically aseptate…………………………… *A. spinosa*

11. Rhizoids generally or rarely septate ……………………………………………12

12. The projections on columellae < 5 μm in length, taper at the end…………………………………………*A. zonata*

12. The projections on columellae > 5 μm in length, rounded at the top……………………*A. pseudocylindrospora*

## Discussion

Phylogenetically, the ITS (Figure 1) and LSU (Figure 2) trees show that four new species cluster in different clades of *Absidia*. The *A. globospora* (100/100/1.00 for both ITS and LSU) is located in the globospora clade and most closely related to *A. glauca* Hagem (88/100/1.00 for ITS). Their sibling relationship is completely supported by ITS and LSU, with 99/100/1.00 and 100/100/1.00 support values, respectively. Physiologically, *A. globospora* is similar to *A. glauca* in heterothallism but differs in maximum growth temperature (37 vs. 29°C). Morphologically, *A. globospora* is similar to *A. glauca* in forming green colonies and globose sporangiospores. However, *A. glauca* differs in its wider sporangiophores (up to 12 μm), glaucous stolons, and larger sporangia (mostly 50- to 60-μm diameter, Ellis and Hesseltine, 1965).

The other three new species are placed in the cylindrospora clade where *A. zonata* is most closely related to *A. koreana* (100/100/1.00 for ITS, 99/95/1.00 for LSU), which is strongly supported with a high value of 100/100/1.00 or 98/99/0.99. Both *A. zonata* and *A. koreana* are physiologically similar in maximum growth temperatures but morphologically differentiated by characteristics on SMA media (Table 2; Ariyawansa et al., 2015). The width of sporangiospores in *A. koreana* are more narrow and uniform. In its protologue, *A. koreana* did not form projections, but the figure in the original article illustrated projections. *A. turgida* is basal to *A. heterospora* in ITS tree (Figure 1) or next to *A. repens* Tiegh. and *A. pararepens* in the LSU tree (Figure 2). *A. medulla* is closely related to *A. repens* in ITS tree (Figure 1) or *A. saloaensis* and *A. ovalispora* in LSU tree (Figure 2).

**TABLE 2:** Comparisons of morphological characteristics of *Absidia zonata* and *Absidia koreana* on SMA media at 25°C.

**Characteristics**	***A. zonata***	***A. koreana***
Colonies	5.5 cm after 4 days	6.2–6.5 cm after 4 days, reverse irregularly zonate
Sporangiophores	1–5 per whorl, occasionally simple (2.6–) 3.2 – 5.6 (–6.5) μm wide	1–6 per whorl, occasionally branched, 3.8–4.6 μm wide
Sporangia	Globose to pyriform, 15.8 – 28.5 (–33.5) × 15 – 25.5 (–31.0) μm	Globose to slightly elliptical, 19.3–23.6 × 21.1–26.4 μm
Columellae	Hemispherical, 11.6–19.6 × 8.4–15.0	Globose, 10.9–17.0 × 11.5–18.9 μm
Sporangiospores	Cylindrical, 3.3–4.5 (–5.0) × 2.1–3.2 (–3.4) μm	Short-cylindrical or cylindrical, 3.5–4.5 × 2.2–2.4 μm
Collars	Present or absent, distinct if presence	Present
Distance from apophyses to septa	(14.2–) 15.2–22.0 (–25.5) μm	17.7–23.5 μm

The two strains, CGMCC 3.16034 and CGMCC 3.16037, of *A. medulla* are similar in maximum growth temperature, micromorphology, and even colonies on MEA and PDA media, but slightly different in colonies on SMA media. The ex-paratype CGMCC 3.16037 is more floccose and thicker and grows more slowly than the ex-holotype CGMCC 3.16034 when they are incubated on SMA at 27°C, and it lacks white concentric rings from the reverse side of the colony (Figure 7).

The species *Chlamydoabsidia padenii* Hesselt. & J.J. Ellis and *Halteromyces radiatus* Shipton & Schipper are obviously nested within *Absidia* in LSU tree (Figure 2). However, morphologically unique multiseptate, easily pigmented aerial chlamydospores were developed in *C. padenii*, whereas dumbbell-shaped sporangia were formed in *H. radiatus* (Hesseltine and Ellis, 1966; Shipton and Schipper, 1975).

The genus *Absidia* was proposed to be divided into several groups distinguishable by their sporangiospores (Kwaśna et al., 2006; Hoffmann et al., 2007, 2009b; Hoffmann and Voigt, 2008; Hoffmann, 2010), which is confirmed in the present study with three well-supported clades, i.e., cylindrospora clade, globospora clade, and cuneospora clade (Figures 1, 2). Two exceptions are worth noting, specifically, both *A. pararepens* and *A. bonitoensis* have sub-globose to globose sporangiospores, even though they are in the cylindrospora clade (Crous et al., 2020).

## Data Availability Statement

The datasets presented in this study can be found in online repositories. The names of the repository/repositories and accession number(s) can be found in the article/supplementary material.

## Author Contributions

T-KZ, C-LZ, and X-YL contributed to conception and design of the study. T-KZ wrote the draft of the manuscript. C-LZ and X-YL improved the manuscript. T-KZ, HZ, C-LZ, and X-YL observed and described the morphology. X-LL and L-YR collected the molecular data. All authors contributed to manuscript revision, proofread, and approved the submitted version.

## Conflict of Interest

The authors declare that the research was conducted in the absence of any commercial or financial relationships that could be construed as a potential conflict of interest.

## Publisher’s Note

All claims expressed in this article are solely those of the authors and do not necessarily represent those of their affiliated organizations, or those of the publisher, the editors and the reviewers. Any product that may be evaluated in this article, or claim that may be made by its manufacturer, is not guaranteed or endorsed by the publisher.

## Footnotes

1
http://www.indexfungorum.org

2
http://nmdc.cn/

3
http://www.geneious.com

## References

[B1] Ariyawansa H. A. Hyde K. D. Jayasiri S. C. Buyck B. Chethana K. T. Dai D. Q. (2015). Fungal diversity notes 111–252—taxonomic and phylogenetic contributions to fungal taxa. *Fungal Divers.* 75 27–274. 10.1007/s13225-015-0346-5

[B2] Benny G. L. (2008). Methods used by Dr. RK Benjamin, and other mycologists, to isolate zygomycetes. *Aliso* 26 37–61. 10.5642/aliso.20082601.08

[B3] Chen J. Fan F. Qu G. Tang J. Xi Y. Bi C. (2020). Identification of *Absidia orchidis* steroid 11β-hydroxylation system and its application in engineering *Saccharomyces cerevisiae* for one-step biotransformation to produce hydrocortisone. *Metab. Eng.* 57 31–42. 10.1016/j.ymben.2019.10.006 31669370

[B4] Cordeiro T. R. L. Nguyen T. T. T. Lima D. X. Silva S. B. G. D. Lima C. F. D. Leitão J. D. (2020). Two new species of the industrially relevant genus *Absidia* (*Mucorales*) from soil of the Brazilian Atlantic Forest. *Acta Bot. Bras.* 34 549–558. 10.1590/0102-33062020abb0040

[B5] Crous P. W. Luangsa-Ard J. J. Wingfield M. J. Carnegie A. J. Hernández-Restrepo M. Lombard L. (2018). Fungal Planet description sheets: 785–867. *Persoonia* 41 238–417. 10.3767/persoonia.2018.41.12 30728607 PMC6344811

[B6] Crous P. W. Wingfield M. J. Chooi Y. H. Gilchrist C. L. Lacey E. Pitt J. I. (2020). Fungal Planet description sheets: 1042–1111. *Persoonia* 44 301–459. 10.3767/persoonia.2020.44.11 33116344 PMC7567971

[B7] de Lima C. L. F. Lima D. X. Cordeiro T. R. L. Lee H. B. Nguyen T. T. T. Gurgel L. M. S. (2021). *Absidia* bonitoensis (*Mucorales*, Mucoromycota), a new species isolated from the soil of an upland Atlantic forest in Northeastern Brazil. *Nova Hedwigia* 112 241–251. 10.1127/nova_hedwigia/2021/0614

[B8] Edler D. Klein J. Antonelli A. Silvestro D. (2020). raxmlGUI 2.0: a graphical interface and toolkit for phylogenetic analyses using RAxML. *Methods Ecol. Evol.* [Preprint]. 10.1101/800912

[B9] Ellis J. J. Hesseltine C. W. (1965). The genus *Absidia*: globose-spored species. *Mycologia* 57 222–235. 10.1080/00275514.1965.12018205

[B10] Ellis J. J. Hesseltine C. W. (1966). Species of *Absidia* with ovoid sporangiospores. II. *Sabouraudia* 5 59–77. 10.1080/00362176785190111 5963263

[B11] Felsenstein J. (1985). Confidence limits on phylogenetics: an approach using bootstrap. *Evolution* 39 783–791. 10.1111/j.1558-5646.1985.tb00420.x 28561359

[B12] Hawksworth D. L. Kirk P. M. Sutton B. C. Pegler D. N. (1995). *Ainsworth & Bisby’s Dictionary of the Fungi*, 8th Edn. UK: CABI Publishing.

[B13] Hesseltine C. W. Ellis J. J. (1961). Notes on *Mucorales*, especially *Absidia*. *Mycologia* 53 406–426. 10.1080/00275514.1961.12017970

[B14] Hesseltine C. W. Ellis J. J. (1964). The genus *Absidia*: *Gongronella* and cylindrical-spored species of *Absidia*. *Mycologia* 56 568–601. 10.1080/00275514.1964.12018145

[B15] Hesseltine C. W. Ellis J. J. (1966). Species of *Absidia* with ovoid sporangiospores. I. *Mycologia* 58 761–785. 10.1080/00275514.1966.12018369 5963263

[B16] Hoffmann K. (2010). “Identification of the genus *Absidia* (Mucorales, Zygomycetes): a comprehensive taxonomic revision,” in *Molecular Identification of Fungi*, eds Gherbawy Y. Voigt K. (Berlin: Springer).

[B17] Hoffmann K. Voigt K. (2008). *Absidia* parricida plays a dominant role in biotrophic fusion parasitism among mucoralean fungi (Zygomycetes): *Lentamyces*, a new genus for *A. parricida* and *A. zychae*. *Plant Biol.* 11 537–554. 10.1111/j.1438-8677.2008.00145.x 19538392

[B18] Hoffmann K. Discher S. Voigt K. (2007). Revision of the genus *Absidia* (*Mucorales*, Zygomycetes) based on physiological, phylogenetic, and morphological characters; thermotolerant *Absidia* spp. form a coherent group, Mycocladiaceae fam. nov. *Mycol. Res.* 111 1169–1183. 10.1016/j.mycres.2007.07.002 17997297

[B19] Hoffmann K. Telle S. Walther G. Eckart M. Kirchmair M. Prillinger H. (2009b). “Diversity, genotypic identification, ultrastructural and phylogenetic characterization of zygomycetes from different ecological habitats and climatic regions: limitations and utility of nuclear ribosomal DNA barcode markers,” in *Current Advances in Molecular Mycology*, eds Gherbawy Y. Mach R. Rai M. (New York, NY: Nova Science Publishers, Inc), 263–312.

[B20] Hoffmann K. Walther G. Voigt K. (2009a). *Mycocladus* vs. *Lichtheimia*: a correction (Lichtheimiaceae fam. nov., *Mucorales*, Mucoromycotina). *Mycol. Res.* 113 275–278.

[B21] Kaczmarek M. B. Struszczyk-Swita K. Li X. Szczêsna-Antczak M. Daroch M. (2019). Enzymatic modifications of chitin, chitosan, and chitooligosaccharides. *Front. Bioeng. Biotech.* 7:243. 10.3389/fbioe.2019.00243 31612131 PMC6776590

[B22] Kwaśna H. Elaine W. Bateman G. L. (2006). Phylogenetic relationships among Zygomycetes from soil based on ITS1/2 rDNA sequences. *Mycol. Res.* 110 501–510. 10.1016/j.mycres.2006.02.004 16769506

[B23] Larsson A. (2014). AliView: a fast and lightweight alignment viewer and editor for large datasets. *Bioinformatics* 30 3276–3278. 10.1093/bioinformatics/btu531 25095880 PMC4221126

[B24] Li G. J. Hyde K. D. Zhao R. L. Hongsanan S. Abdel-Aziz F. A. Abdel-Wahab M. A. (2016). Fungal diversity notes 253–366: taxonomic and phylogenetic contributions to fungal taxa. *Fungal Divers.* 78 1–237. 10.1007/s13225-016-0366-9

[B25] Lima D. X. Cordeiro T. R. De Souza C. A. De Oliveira R. J. Lee H. B. Souza-Motta C. M. (2020). Morphological and molecular evidence for two new species of *Absidia* from Neotropic soil. *Phytotaxa* 446 61–71. 10.11646/phytotaxa.446.1.8

[B26] Orr G. F. Plunkett O. A. (1959). A new species of *Absidia* from California. *Mycologia* 51 203–209. 10.1080/00275514.1959.12024813

[B27] Ridgway R. (1912). *Color Standards and Color Nomenclature.* Washington, DC: Ridgway.

[B28] Ronquist F. Teslenko M. Van Der Mark P. Ayres D. L. Darling A. Höhna S. (2012). MrBayes 3.2: efficient Bayesian phylogenetic inference and model choice across a large model space. *Syst. Boil.* 61 539–542. 10.1093/sysbio/sys029 22357727 PMC3329765

[B29] Schipper M. A. A. (1990). Notes on *Mucorales* – I. Observations on *Absidia*. *Persoonia* 14 133–149.

[B30] Shipton W. A. Schipper M. A. (1975). *Halteromyces*, a new genus in the *Mucorales*. *Antonie van Leeuwenhoek* 41 337–342. 10.1007/BF02565068 1082298

[B31] Swofford D. L. (2002). *PAUP^∗^: Phylogenetic Analysis Using Parsimony (^∗^and Other Methods). Version 4.0b10.* Sunderland, MA: Sinauer Associates.

[B32] van Tieghem P. (1876). Troisième mémoire sur les Mucorinées. *Annales des Siences Naturelles Botanique* 4 312–399.

[B33] Wanasinghe D. N. Phukhamsakda C. Hyde K. D. Jeewon R. Lee H. B. Jones E. G. (2018). Fungal diversity notes 709–839: taxonomic and phylogenetic contributions to fungal taxa with an emphasis on fungi on *Rosaceae*. *Fungal Divers.* 89 1–236. 10.1007/s13225-018-0395-7

[B34] Wang Y. N. Liu X. Y. Zheng R. Y. (2014). Umbelopsis changbaiensis sp. nov. from China and the typification of *Mortierella vinacea*. *Mycol. Prog.* 13 657–669. 10.1007/s11557-013-0948-9

[B35] White T. J. Bruns T. Lee S. Taylor J. (1990). “Amplification and direct sequencing of fungal ribosomal RNA genes for phylogenetics,” in *PCR Protocols: a Guide to Methods and Applications*, eds Innis M. A. Gelfand D. H. Sninsky J. J. White T. J. (San Diego, CA: Academic Press), 315–322. 10.1016/b978-0-12-372180-8.50042-1

[B36] Zhang T. Y. Yu Y. Zhu H. Yang S. Z. Yang T. M. Zhang M. Y. (2018). *Absidia* panacisoli sp. nov., isolated from rhizosphere of *Panax notoginseng*. *Int. J. Syst. Evol. Microbiol.* 68 2468–2472. 10.1099/ijsem.0.002857 29927367

[B37] Zhao C. L. Wu Z. Q. (2017). *Ceriporiopsis* kunmingensis sp. nov. (*Polyporales*, Basidiomycota) evidenced by morphological characters and phylogenetic analysis. *Mycol. Prog.* 16 93–100. 10.1007/s11557-016-1259-8

[B38] Zhao H. Zhu J. Zong T. K. Liu X. L. Ren L. Y. Lin Q. (2021). Two New Species in the Family Cunninghamellaceae from China. *Mycobiology* 49 142–150. 10.1080/12298093.2021.1904555 PMC10635138 37970189

[B39] Zheng R. Y. Chen G. Q. (2001). A monograph of *Cunninghamella*. *Mycotaxon* 80 1–75. 10.1007/978-3-319-23534-9_1

[B40] Zheng R. Y. Liu X. Y. (2018). *Species Catalogue of China. Volume 3. Fungi: Chtrid, Zygomycotan, Glomeromycotan Fungi.* Beijing: Science Press.

